# Conduction in ulnar nerve bundles that innervate the proximal and distal muscles: a clinical trial

**DOI:** 10.1186/1471-2377-10-81

**Published:** 2010-09-13

**Authors:** Attila Oğuzhanoğlu, Sibel Güler, Mustafa Çam, Eylem Değirmenci

**Affiliations:** 1 Pamukkale University, School of Medicine, Department of Neurology, Araştırma Hastanesi, Kınıklı-Denizli, Turkey; 2 Devlet Hastanesi, Siirt, Turkey; 3 Asker Hastanesi, Eskişehir, Turkey

## Abstract

**Background:**

This study aims to investigate and compare the conduction parameters of nerve bundles in the ulnar nerve that innervates the forearm muscles and hand muscles; routine electromyography study merely evaluates the nerve segment of distal (hand) muscles.

**Methods:**

An electrophysiological evaluation, consisting of velocities, amplitudes, and durations of ulnar nerve bundles to 2 forearm muscles and the hypothenar muscles was performed on the same humeral segment.

**Results:**

The velocities and durations of the compound muscle action potential (CMAP) of the ulnar nerve bundle to the proximal muscles were greater than to distal muscles, but the amplitudes were smaller.

**Conclusions:**

Bundles in the ulnar nerve of proximal muscles have larger neuronal bodies and thicker nerve fibers than those in the same nerve in distal muscles, and their conduction velocities are higher. The CMAPs of proximal muscles also have smaller amplitudes and greater durations. These findings can be attributed to the desynchronization that is caused by a wider range of distribution in nerve fiber diameters.

Conduction parameters of nerve fibers with different diameters in the same peripheral nerve can be estimated.

## Background

Peripheral motor nerve diameter decreases gradually after emerging from the spinal cord toward the target muscles. In a myelinated nerve fiber, the thickness of the fiber correlates positively with nerve conduction velocity; conduction velocity declines when a fiber's diameter decreases[1].

Nerve diameter is proportional to the size of the motor nerve body in the anterior horn [1]. Nerve diameter thickness and conduction velocity correlate with nerve body size. Proximal muscles with bigger masses are innervated by thicker fibers [2-5].

In addition to the sciatic nerve [4,6], the nerve velocities in the ulnar nerve can be recorded and calculated separately between the proximal and distal muscles of the upper extremities. Thus, one can differentiate between the fastest conductive fibers that innervate the proximal and distal muscles electrophysiologically.

In this study, we examined the nerve conduction velocities, compound muscle action potential (CMAP) amplitudes, and duration of 2 proximally positioned forearm muscles that have greater mass and hypothenar muscles that are distally positioned with smaller mass.

## Methods

This study was performed using cases that were referred for evaluation in the EMG laboratory (Premiere Plus EMG Device. Medelec/Vickers Medical, Manor Way, Old Woking, Surrey, United Kingdom, GU22 9JU) and was approved by the Local Ethical Committee (Clinical Researches Committee, Denizli Province, Ministry of Health, Republic of Turkey). All patients gave informed consent. The patients were included after undergoing a routine electrophysiological protocol to exclude polyneuropathy and any neuropathy. The study was performed in the right upper extremity in all normative subjects (30 subjects: 6 men, 24 women). The mean (±SD) age of the patients was 38.7 (±15.4) (Range 16-70). Room temperature always exceeded 24°C. The subject was lain down. The right arm was positioned 70-90 degrees to the body, and the forearm lay 90 degrees to the arm; this position was maintained throughout the study.

### Stimulus

1. Ulnar nerve stimuli in the arm segment: A point, 5 cm proximal to the medial epicondyle, was selected as the distal stimulus point. A point, 12 cm proximal to the distal point in the axillary region, was the proximal stimulus point.

2. Ulnar nerve stimuli in the forearm segment: A point, 5 cm distal to the medial epicondyle, was chosen as the proximal stimulus point. A point, 5 cm proximal to the distal wrist line, served as the distal stimulus point.

The supramaximal level of the stimulus intensity was increased slowly until a point was reached at which the CMAP amplitude no longer increased. The point at which it rose 25% more to ensure that the amplitude did not change further was selected as the severity of stimulus in all cases, and the stimulus duration was 100 μs.

### Recording

#### 1 - Forearm recording

A-Recording from the flexor carpi ulnaris muscle (FCU): The active disc electrode (silver, 10 mm in diameter) was positioned at a point 2 digits wide from the ulna, where the proximal third and medial third sections of the forearm met. The reference electrode was positioned on the ulna, transverse to the active electrode [7,8].

B - Recording from the flexor digitorum profundus muscle (FDP): The recording was made by a bar electrode (40 mm in length, 20 × 8 mm recording surface for both the anode and cathode); the active part was positioned on the medial third of the forearm, near the ulna, and the reference was positioned distally [7].

#### 2 - Hand (Hypothenar-HYT, abductor digiti minimi muscle-ADM) recording

The active bar electrode was positioned at the midpoint between the wrist distal line and the metacarpophalangeal joint [8]. The reference bar electrode was positioned on the metacarpophalangeal joint, a more distal position.

The screen sweep time was 30 ms, and the sensitivity was 2-5 mV.

### Study scheme

After the subject lay down and the right upper extremity was positioned as discussed, the FCU, FDP, and HYT muscles were recorded alternatively by stimulating the arm segment separately for each recording. Then, a recording of the HYT muscle was made by stimulating the forearm. Thus, nerve conduction velocities and CMAP amplitude and duration were measured in the 3 responses from 3 different muscles in the arm and hand. Amplitude was measured from onset to the negative peak, and duration was measured from the onset of the first negative deflection to the last point at which the potential returned to baseline. Latency was measured from the stimulus artefact to the onset of negative deflection. Additionally, nerve conduction velocity and CMAP amplitude and duration in the forearm segment was measured in the responses from the ADM (HYT) muscle.

### Statistics

Kolmogorov-Smirnov test was performed for the appropriateness of data distribution to the normal distribution. Because all data were distributed normally, we used t-test in paired groups and Bonferroni correction for data comparisons and Pearson's correlation analysis to determine the correlation between the data. p < .05 was the significance level. SPSS v.16 was used for all statistical evaluations.

## Results

### Ulnar nerve conduction velocities

Velocities from 2 forearm muscles (FCU, FDP) and 1 hand muscle (HYP) after stimulation of the arm segment were compared. These velocities were also compared with that obtained of the HYP area after stimulation of the forearm segment. The comparison of these velocities are shown in Table 1.

**Table 1:** Nerve conduction velocities after stimulus on the arm and forearm segments

Velocity	Mean (±SD) (m/sn)	n	Comparison	T value	P
V_FCU_	76.27 (12.67)	30	V_FCU - _V_FDP_	-0.41	0.685
V_FDP_	76.25 (13.36)	30	V_FCU - _V_HYP_	5.61	0.000
V_HYP_	61.90 (7.21)	30	V_FCU - _V_HYP-FA_	6.32	0.000
V_HYP-FA_	62.26 (5.05)	30	V_FDP - _V_HYP_	5.87	0.000
			V_FDP - _V_HYP-FA_	5.44	0.000
			V_HYP - _V_HYP-FA_	-0.26	0.793

m: meter, s: second

V_FCU_: Ulnar nerve conduction velocity in the FCU muscle

V_FDP_: Ulnar nerve conduction velocity in the FDP muscle

V_HYP_: Ulnar nerve conduction velocity in the HYP area muscle after stimuli on the arm segment

V_HYP-FA_: Ulnar nerve conduction velocity in HYP area muscles after stimuli on the forearm segment

The velocities V_FCU _and V_FDP _in the arm segment did not differ, as calculated by the responses from both forearm muscles.

The velocities in the hypothenar area did not differ between the arm segment (V_HYP_) and forearm segment (V_HYP-FA_).

Both velocities of the arm segment (V_FCU_, V_FDP_) from the forearm muscles exceeded those from the arm segment (V_HYP_) and forearm segment (V_HYP-FA_), both of which were recorded from the hypothenar area.

### Compound muscle action potentials

#### A-Amplitude

In the CMAP evaluation, comparisons were made by calculating the average CMAP amplitudes, obtained after proximal and distal stimulation of each muscle. CMAP amplitudes of 2 forearm muscles (AMP_FCU_, AMP_FDP_) and 1 hand muscle (AMP_HYP_) after stimulation of the arm segment were compared with the amplitude (AMP_HYP-FA_) from the HYP area after stimulation of the forearm segment. These comparisons are shown in Table 2.

**Table 2:** CMAP amplitudes after stimulus on the arm and forearm segments.

Amplitude	Mean (±SD) (mV)	n	Comparison	T value	P
AMP_FCU_	3.28 (1.33)	30	AMP_FCU - _AMP_FDP_	-0.43	0.670
AMP_FDP_	3.17 (1.48)	30	AMP_FCU - _AMP_HYP_	-6.83	0.000
AMP_HYP_	5.87 (1.81)	30	AMP_FCU - _AMP_HYP-FA_	-8.51	0.000
AMP_HYP-FA_	6.47 (2.06)	30	AMP_FDP - _AMP_HYP_	-7.38	0.000
			AMP_FDP - _AMP_HYP-FA_	-8.89	0.000
			AMP_HYP _- AMP_HYP-FA_	-3.55	0.001

mV:millivolt

AMP_FCU_: CMAP amplitude in the FCU muscle

AMP_FDP_: CMAP amplitude in the FDP muscle

AMP_HYP_: CMAP amplitude in the HYP area muscle after stimulus on the arm segment

AMP_HYP-FA_: CMAP amplitude in the HYP area muscles after stimulus on the forearm segment

The CMAP amplitudes of the forearm muscles (AMP_FCU_, AMP_FDP_) did not differ.

The CMAP amplitudes from both forearm muscles (AMP_FCU_, AMP_FDP_) were lower than the 2 amplitudes of the hypothenar (AMP_HYP _and AMP_HYP-FA_).

The amplitude in the hypothenar area (AMP_HYP_) was lower compared with that of the forearm (AMP_HYP-FA_) segment.

#### B - Response duration

We compared the average CMAP duration after proximal and distal stimulation. CMAP durations from the 2 forearm muscles (DUR_FCU_, DUR_FDP_) and 1 hand muscle (DUR_HYP_) after stimulation of the arm segment were compared with that from the HYP area after stimulation of the forearm segment. This comparison is shown in Table 3.

**Table 3:** CMAP duration after stimulus on the arm and forearm segments.

Duration	Mean (±SD) (ms)	n	Comparison	T value	P
DUR_FCU_	7.45 (1.26)	30	DUR_FCU - _DUR_FDP_	-1.87	0.071
DUR_FDP_	7.97 (1.37)	30	DUR_FCU - _DUR_HYP_	-5.53	0.000
DUR_HYP_	5.90 (1.04)	30	DUR_FCU - _DUR_HYP-FA_	7.44	0.000
DUR_HYP-FA_	5.76 (0.88)	30	DUR_FDP - _DUR_HYP_	-7.38	0.000
			DUR_FDP - _DUR_HYP-FA_	7.96	0.000
			DUR_HYP - _DUR_HYP-FA_	0.68	0.504

ms: millisecond

DUR_FCU_: CMAP duration in the FCU muscle

DUR_FDP_: CMAP duration in the FDP muscle

DUR_HYP_: CMAP duration in the HYP area muscle after stimulus on the arm segment

DUR_HYP-FA_: CMAP duration in the HYP area muscles after stimulus on the forearm segment

We observed no difference in CMAP duration between the forearm muscles (DUR_FCU_, DUR_FDP_).

CMAP duration in the hypothenar area (DUR_HYP_) and forearm (DUR_HYP-FA_) did not differ.

The CMAP duration in both forearm muscles (DUR_FCU_, DUR_FDP_) was longer than that in the hypothenar areas (DUR_HYP _and DUR_HYP-FA_).

#### Correlation between average velocity, amplitude, and duration

The correlation between the 4 groups of velocities, amplitudes, and durations is shown in Table 4. A strong negative correlation from arm to forearm changes was observed between velocity and amplitude and between amplitude and duration. In addition, velocity and duration had a strong positive correlation.

**Table 4:** Correlations between velocity, amplitude, and duration.*

Variables	r	P
Velocity-Amplitude	-0.987	0.013
Velocity-Duration	0.986	0.014
Amplitude-Duration	-0.982	0.018

*: Pearson's correlation analysis. r:correlation coefficient.

## Discussion

In studies in the hind limb of rats [4] and lower extremities in humans [6], n. tibialis fibers that extend to the m. gastrocnemius, which is a proximal muscle, conduct faster than the tibial nerve fibers that connect to interosseous muscles, the small muscles of the feet.

Cullheim [1] has shown that motor neuron size in the anterior horn correlates positively with intramedullary axon diameter and axon conduction velocity and that the correlation between the first axon segment and axon conduction velocity is the most powerful one.

In their study on the hind limb in mouse, McHanwell and Biscoe [3] examined motor neuron body areas and showed that body areas of the nerves that travel to proximally positioned femoral muscles are larger than those of distally positioned crucial muscles. The histograms of body area of proximally positioned muscles are bimodal, and those of the distal foot muscles are unimodal.

Fernand and Young [9] demonstrated that nerves of proximally positioned muscles are thicker than those of distally positioned muscles in the upper and lower extremities in rabbit. Histograms of the diameters of proximal muscles nerves show a bimodal distribution, while those of distal foot interosseous muscles are unimodal. They noted in their classification of nerve fibers that a considerable percentage of bimodally distributed nerve fibers exceeded 14 μm in diameter and can reach 24 μm and that a minute proportion of unimodal muscular fibers are 10-12 μm; most of them, however, fall below these values.

Devanandan et al. [10] noted a similarity and unimodal distribution between histograms of the profound branch of the n. ulnaris in Bonnet monkeys (Macaca radiata) and the profound branch of the human n. ulnaris. Most hand muscles (Flexor digiti minimi, opponens digiti minimi, adductor pollicis, and the first dorsal interosseal muscles) showed unimodal distribution; the abductor digiti minimi showed bimodal distribution. Furthermore, the authors emphasized that none of the nerve fibers in these muscles exceeded 12 μm.

Buchtal and Schmalbruch [2] has suggested that with regard to motor unit size, conduction velocity changes according to the size of the motor neuron and muscle mass; because proximally positioned muscles are larger in mass, they are believed to have larger motor neurons.

This is the first study that compares conduction velocities in fibers that enter the ulnar nerve and reach 2 disparate muscle groups in the arm and hand segments. It has been shown electrophysiologically that nerve fibers that innervate 2 muscles that are proximally positioned and larger in mass (FCU and FDP) conduct faster than those that innervate distal muscles that are smaller. This finding indicates that nerve fibers of the proximal muscles are thicker in the arm segment.

Nerves branch and become thinner conically after emerging from the spinal cord during their march toward the muscles, becoming even thinner [9]. Because the conduction velocity of the proximally and distally positioned muscles was measured in the same arm segment in this study, we propose that the difference between the velocities does not depend on proximodistal thinning of the nerve diameter. Based on our results, conduction velocities are higher in fibers of the ulnar nerve of proximal muscles (FCU, FDP), which have larger neuronal bodies and thicker nerve fibers than fibers in the same nerve of the distal muscles (HYP).

No difference was observed between the conduction velocities in the arm and forearm segments by hypothenar recording, but data are conflicting on this subject. The chief problem is whether the velocity in the elbow segment is included in the proximal or distal segment velocity. Harding and Halar [11] have opined how ulnar nerve conduction is influenced by elbow angle in humans and cadavers, demonstrating that conduction time increases in the forearm and that motor conduction velocity decreases in the forearm segment if the forearm is positioned 45° from the flexion position to extension. They have reported in cadavers that increasing elbow flexion decreases ulnar nerve wrinkledness and that the nerve relocates distally and becomes smoother in the above-elbow segment [11].

Flexion of the forearm affects not only the position of the ulnar nerve in the elbow segment but also the position of the nerve in the arm and forearm segments by shifting the nerve in these segments. It has been suggested that flexion in the elbow beyond 90° does not increase conduction velocity, and recordings in the hypothenar area with the elbow in this position have shown that the velocities in the 3 segments are equal and that 90° flexion is the most suitable position [12]. In our study, with the elbow in 90° flexion, there was no difference between conduction velocities in the arm and forearm segments in the hypothenar recordings, demonstrating that diameter thinning in the nerve fibers that innervate the hypothenar muscles precludes them from reaching to an extent that is sufficient to cause changes in conduction velocity.

In proportion to the distance between the stimulus point and recording point, the sensorial (and also less marked in motor) action potential amplitudes and areas decrease and the response duration climbs[13]. This finding is due temporal dispersion [13-15]. In our study, arm segment amplitudes in the HYT recording were smaller than the forearm segment amplitudes in the HYT recording, and there was no difference between the durations.

Notably, the amplitudes from the forearm muscles (FCU, FDP) were smaller than those in the HYP area, and the durations were longer than the HYP response durations. Differences in temperature have been proposed to explain the disparities between the arm and forearm segments in nerve conduction studies. Although the effect of temperature on nerve conduction velocity has found near-total acceptance, its effect on amplitude has not. Studies have measured temperature differences between arm and forearm segments of 0.6°C [16] and 1.1°C [17]. Both have stated that there is no relation between variations in temperature and conduction velocity[16,17]. Todnem et al. [18] have suggested that the median nerve motor amplitude does not undergo significant alterations with temperature changes.

We need to find factors other than temperature that explain the differences in amplitude between proximal and distal muscles in this study. Fernand and Young [9] reported that nerve diameters of proximal muscles show bimodal distribution in rabbits, exceeding those of nerves in distal muscles, which had unimodally distributed nerve fibers. They also found that nerve fibers of muscles with unimodal distribution had primarily the same diameter, failing to observe nerve fibers that had large or tiny diameters. There are fibers that have very small and very large diameters, and nerves in proximal forearm muscles have a wide range of diameters. Based on the results of Devanandan et al.[10] and our study, we postulate that nerve fibers of the FCU and FDP muscles are bimodal and that nerve fibers of HYP area muscles are unimodally distributed, assuming that similar features exist in humans.

Proximal muscles should have nerve fibers with a wide range of diameters, and distal muscles should have a narrower range of nerve fiber diameters. For example, nerve fibers in the m. semimembranosus, a proximal muscle in the posterior extremity in rabbit, have diameters that shift between 2-20 μm, and those of nerve fibers in the m. interosseous, a distal muscle, are between 2-12 μm [9]. In our study, the reduction in CMAP amplitude and prolongation in response duration in the forearm proximal muscles might be caused by the desynchronization that arises from the magnitude of the temporal dispersion that is caused by differences between conduction velocities of their nerve fibers. This situation is comparable with the desynchronization that is caused increased stimulus distances - the result of temporal dispersion during recording in the HYP region [14,15].

With regard to the relationship between parameters of nerve conduction velocities, velocity and response duration correlated positively, and a negative correlation was observed between velocity and amplitude and between amplitude and duration, all of which were strong. Nerve conduction velocity decreased from the proximal to the distal, as did duration, and amplitude increased (Table 4).

## Conclusion

Nerve bundles that travel to proximally positioned muscles (FCU, FDP) that are innervated by the ulnar nerve have higher nerve conduction velocities compared with those of bundles that extend to distally positioned muscles (HYP). The CMAP amplitudes of proximal muscles are lower than those of distal muscles, and their duration is longer. This finding is attributed to the desynchronization that is caused by the wide range in diameters of nerve fibers in the proximal muscles, arising during nerve stimulation and after temporal dispersion. Although our sample size was not high, we believe that separate EMG recordings from proximal and distal muscles might yield insights into nerve fibers and size of motor nerve cell bodies.

## Abbreviations

EMG: Electromyography; CMAP: compound muscle action potential; FCU: flexor carpi ulnaris muscle; FDP: flexor digitorum profundus muscle; HYT: Hypotenar area; ADM: abductor digiti minimi muscle; AMP_FCU_: CMAP amplitude in the FCU muscle; AMP_FDP_: CMAP amplitude in the FDP muscle; AMP_HYP_: CMAP amplitude in the HYP area muscle after stimulus on the arm segment; AMP_HYP-FA_: CMAP amplitude in the HYP area muscles after stimulus on the forearm segment; DUR_FCU_: CMAP duration in the FCU muscle; DUR_FDP_: CMAP duration in the FDP muscle; DUR_HYP_: CMAP duration in the HYP area muscle after stimulus on the arm segment; DUR_HYP-FA_: CMAP duration in the HYP area muscles after stimulus on the forearm segment; SPSS: Statistical Package for the Social Sciences; V_FCU_: Ulnar nerve conduction velocity in the FCU muscle; V_FDP_: Ulnar nerve conduction velocity in the FDP muscle; V_HYP_: Ulnar nerve conduction velocity in the HYP area muscle after stimuli on the arm segment; V_HYP-FA_: Ulnar nerve conduction velocity in HYP area muscles after stimuli on the forearm segment; Cm: centimeter; m: meter; mV: milivolt; s: second; SD: Standard deviation; μm: micrometer; μs: microsecond; ° C: degree Celcius

## Competing interests

The authors declare that they have no competing interests.

## Authors' contributions

SG and MÇ helped to perform EMG studies and to collect data. ED helped to translate into English and revised manuscript. AO designed study, performed EMG studies, collected data and wrote the article. All authors read and approved the final manuscript.

## Authors' information

All authors are specialists in neurology. Additionally, AO is a professor in neurology and ED is an assistant professor in neurology.

## Pre-publication history

The pre-publication history for this paper can be accessed here:

http://www.biomedcentral.com/1471-2377/10/81/prepub

## References

[B1] Cullheim S Relations between cell body size, axon diameter and axon conduction velocity of cat sciatic α-motoneurons stained with horseradish peroxidase Neurosci Lett 1978 8 17 20 10.1016/0304-3940(78)90090-3 19605142

[B2] Buchthal F Schmalbruch H Motor unit of mammalian muscle Physiol Rev 1980 60 90 142 6766557 10.1152/physrev.1980.60.1.90

[B3] McHanwell S Biscoe TJ The sizes of motoneurons supplying hindlimb muscles in the mouse Proc R Soc Lond B Biol Sci 1981 213 201 16 10.1098/rspb.1981.0062 6120515

[B4] Oğuzhanoğlu A Erdoğan Ç Tabak E Cenikli U Comparison of conduction velocities of nerve fibers to smaller and larger muscles in rats Int J Neurosci 2010 120 76 79 10.3109/00207450903389370 20128676

[B5] Henneman E Olson CB Relations between structure and function in the design of skeletal muscles J Neurophysiol 1965 28 581 598 14328455 10.1152/jn.1965.28.3.581

[B6] Gassel MM Trojaborg W Clinical and electrophysiological study of the pattern of conduction times in the distribution of the sciatic nerve J Neurol Neurosurg Psychiatry 1964 27 351 7 10.1136/jnnp.27.4.351 14200791 PMC495758

[B7] Felsenthal G Brockman PS Mondell DL Hilton EB Proximal forearm ulnar nerve conduction techniques Arch Phys Med Rehabil 1986 67 440 4 3729688

[B8] Delagi EF Perotto A Anatomic Guide for Electromyographer 1980 2 Springfield, IL, Thomas

[B9] Fernand VS Young JZ The sizes of the nerve fibres of muscle nerves Proc R Soc Lond B Biol Sci 1951 139 38 58 10.1098/rspb.1951.0045 14911813

[B10] Devanandan MS Ghosh S Simoes EA Myelinated fibers of the deep branch of the ulnar nerve at the wrist in bonnet monkeys (Macaca radiata) and some of its branches to the hand Anat Rec 1980 197 387 96 10.1002/ar.1091970403 7212292

[B11] Harding C Halar E Motor and sensory ulnar nerve conduction velocities: effect of elbow position Arch Phys Med Rehabil 1983 64 227 32 6847360

[B12] Buschbacher RM Ulnar nerve motor conduction to the abductor digiti minimi Am J Phys Med Rehabil 1999 78 6 Suppl S9 14 10.1097/00002060-199911001-00003 10573091

[B13] Johnsen B Fuglsang-Frederiksen A de Carvalho M Labarre-Vila A Nix W Schofield I Amplitude, area and duration of the compound muscle action potential change in different ways over the length of the ulnar nerve Clin Neurophysiol 2006 117 2085 92 10.1016/j.clinph.2006.05.033 16876477

[B14] Olney RK Budingen HJ Miller RG The effect of temporal dispersion on compound action potential area in human peripheral nerve Muscle Nerve 1987 10 728 33 10.1002/mus.880100809 3683446

[B15] Kimura J Sakimura Y Machida M Fuchigami Y Ishida T Claus D Effect of desynchronized inputs on compound sensory and muscle action potentials Muscle Nerve 1988 11 694 702 10.1002/mus.880110705 3405238

[B16] Trojaborg W Motor nerve conduction velocities in normal subjects with particular reference to the conduction in proximal and distal segments of median and ulnar nerve Electroencephalogr Clin Neurophysiol 1964 17 314 21 10.1016/0013-4694(64)90132-4 14207699

[B17] Spiegel MH Johnson EW Conduction velocity in the proximal and distal segments of the motor fibers of the ulnar nerve of human beings Arch Phys Med Rehabil 1962 43 57 61 13915757

[B18] Todnem K Knudsen G Riise T Nyland H Aarli JA The non-linear relationship between nerve conduction velocity and skin temperature J Neurol Neurosurg Psychiatry 1989 52 4 497 501 10.1136/jnnp.52.4.497 2738592 PMC1032299

